# Targeting Colorectal Cancer Cells with Niosomes Systems Loaded with Two Anticancer Drugs Models; Comparative In Vitro and Anticancer Studies

**DOI:** 10.3390/ph15070816

**Published:** 2022-06-30

**Authors:** Shaymaa Wagdy El-Far, Hadel A. Abo El-Enin, Ebtsam M. Abdou, Ola Elsayed Nafea, Rehab Abdelmonem

**Affiliations:** 1 Division of Pharmaceutical Microbiology, Department of Pharmaceutics and Industrial Pharmacy, College of Pharmacy, Taif University, P.O. Box 11099, Taif 21944, Saudi Arabia; 2 Department of Pharmaceutics and Industrial Pharmacy, College of Pharmacy, Taif University, P.O. Box 11099, Taif 21944, Saudi Arabia; 3 Department of Pharmaceutics, National Organization of Drug Control and Research (NODCAR), Giza P.O. Box 12511, Egypt; ebtsamabdou83@gmail.com; 4 Department of Clinical Pharmacy, College of Pharmacy, Taif University, P.O. Box 11099, Taif 21944, Saudi Arabia; oenafea@tu.edu.sa; 5 Department of Industrial Pharmacy, College of Pharmaceutical Sciences and Drug Manufacturing, Misr University for Science and Technology (MUST), 6th of October City P.O. Box 12566, Egypt; drrahoba@yahoo.com

**Keywords:** Colorectal Cancer, Niosomes, Oxaliplatin, Paclitaxel, d-α-tocopheryl polyethylene glycol 1000 succinate (TPGS)

## Abstract

Colorectal cancer (CRC) is considered one of the most commonly diagnosed malignant diseases. Recently, there has been an increased focus on using nanotechnology to resolve most of the limitations in conventional chemotherapy. Niosomes have great advantages that overcome the drawbacks associated with other lipid drug delivery systems. They are simple, cheap, and highly stable nanocarriers. This study investigated the effectiveness of using niosomes with their amphiphilic characteristics in the incorporation of both hydrophilic and hydrophobic anticancer drugs for CRC treatment. Methods: Drug-free niosomes were formulated using a response surface D-optimal factorial design to study the cholesterol molar ratio, surfactant molar ratio and surfactant type effect on the particle size and Z-potential of the prepared niosomes. After numerical and statistical optimization, an optimized formulation having a particle size of 194.4 ± 15.5 nm and a Z-potential of 31.8 ± 1.9 mV was selected to be loaded with Oxaliplatin and Paclitaxel separately in different concentrations. The formulations with the highest entrapment efficiency (EE%) were evaluated for their drug release using the dialysis bag method, in vitro antitumor activity on HT-29 colon cancer cell line and apoptosis activity. Results: Niosomes prepared using d-α-tocopheryl polyethylene glycol 1000 succinate (TPGS) at a molar ratio 4, cholesterol (2 molar ratio) and loaded with 1 molar ratio of either Oxaliplatin or Paclitaxel provided nanosized vesicles (278.5 ± 19.7 and 251.6 ± 18.1 nm) with a Z-potential value (32.7 ± 1.01 and 31.69 ± 0.98 mV) with the highest EE% (90.57 ± 2.05 and 93.51 ± 2.97) for Oxaliplatin and Paclitaxel, respectively. These formulations demonstrated up to 48 h drug release and increased the in vitro cytotoxicity and apoptosis efficiency of both drugs up to twice as much as free drugs. Conclusion: These findings suggest that different formulation composition parameters can be adjusted to obtain nanosized niosomal vesicles with an accepted Z-potential. These niosomes could be loaded with either hydrophilic drugs such as Oxaliplatin or hydrophobic drugs such as Paclitaxel. Drug-loaded niosomes, as a unique nanomicellar system, could enhance the cellular uptake of both drugs, resulting in enhanced cytotoxic and apoptosis effects against HT-29 colon cancer cells. Oxaliplatin–niosomes and Paclitaxel–niosomes can be considered promising alternative drug delivery systems with enhanced bioavailability of these two anticancer drugs for colorectal cancer treatment.

## 1. Introduction

Colorectal cancer (CRC) is a serious cancer type that is considered one of the most recently diagnosed malignant diseases. The incidence and mortality rates were higher in men than in women, especially in developed countries. In addition to its high mortality rate, it still ranks fifth in all tumor-related diseases and third in the United States among diagnosed male and female patients [1]. Colorectal cancer primary therapy management is surgery, but in non-metastatic disease (stages I–III), chemotherapy is used as adjuvant therapy in stage II disease and the majority of stage III and in the metastatic colorectal cancer progress patients [2,3].

Oxaliplatin is used for colorectal cancer treatment and could be used in the treatment of other tumors. It is the third-generation organo-platinum compound that could be used as a monotherapy or in combination with 5-fluorouracil (5-FU) for colorectal carcinoma treatment. Oxaliplatin is a monoclonal antibody that targets the epidermal growth factor receptor, triggers the immobilization of the mitotic cell cycle in colorectal tumor cells, and induces apoptosis [4,5]. Oxaliplatin monotherapy for colorectal cancer untreated patients produces response rates of about 12% to 24%, while for relapsed or refractory advanced colorectal cancer patients, it is from 10% to 11% [6].

Oxaliplatin is slightly soluble in water with a narrow therapeutic index drug; therefore, small changes in the dose can greatly affect the clinical efficacy and toxicity [7,8]. Oxaliplatin’s toxicity is the peripheral sensory neuropathy, which is mainly two types. The first is acute sensory neuropathy and is exacerbated by cold temperatures (e.g., laryngopharyngeal dysesthesia), and it is completely reversible. After 24 weeks of Oxaliplatin administration, cumulative and frequent sensory neuropathy occurs. Chronic sensory neuropathy, the second type, slowly reverses after treatment is discontinued, and this side effect represents its dose-limiting toxicity [9]. These limitations of systemic toxicity and lower therapeutic index activity are mainly attributed to the high drug accumulation in erythrocytes compared to the lower drug accumulation in tumor tissues following intravenous administration [10].

Paclitaxel has been reported as an effective chemotherapy in the treatment of colorectal cancer [11]. At low doses, it regulates glutaminolysis, which inhibits tumor cell growth. It inhibits the tumor cells’ proliferation and angiogenesis and enhances apoptosis. The mechanism of action is closely related to its ability to promote the polymerization of tubulin into microtubules by binding microtubules and stabilizing cell division [12,13,14].

The lower oral Paclitaxel bioavailability (<10%) is observed due to efflux of the drug by the multidrug transporter P-glycoprotein (Pgp) and excessive hepatic metabolism by the cytochrome P450 system [15]. In addition, Paclitaxel is highly lipophilic, insoluble in water, and lacks ionizable functional groups; therefore, changing pH does not enhance its solubility, and it cannot be used in a pharmaceutically different form [16].

Recently, there has been an increased focus on using nanotechnology to develop novel and targeted drug delivery systems. The unique properties of the nanosized drug delivery systems that arise from the small-sized particles and the large surface area of the vesicles may lead to improve the drugs’ passive targeting properties. Additionally, the latter helps in maintaining more drug-loaded vesicles into tumor cells by enhancing the permeability and retention effect. They enhance the dose efficacy and reduce the side effects [17] and help in using the chemotherapy at low concentrations [18], which resolves most of the limitations in conventional chemotherapy [19].

Niosomes are a type of nanoparticle drug delivery systems known as non-ionic surfactant vehicles (NSVs). Niosomes act as self-assembly closed spheroidal structures of non-ionic amphiphiles in the aqueous medium [20]. They have the ability to entrap both hydrophilic and hydrophobic drugs in their core and between the bilayers, respectively [21]. Therefore, it is considered a good drug delivery system for many active agents as phytochemicals, extracts, drugs, and many anticancer drugs (e.g., methotrexate, doxorubicin, and cisplatin) [22,23]. Niosomes are considered simple, cheap, and highly stable nanocarriers compared to many other nanocarriers which could be used in treatment and diagnosis in cancer therapy [24]. They have great advantages that overcome the drawbacks associated with other lipid drug delivery systems as liposomes, as they have greater chemical stability, long shelf life, high purity, content uniformity, low cost, and convenient storage [25]. They have the ability to prolong the circulation of entrapped drugs, minimize drug degradation and inactivation after administration, which helps in preventing undesirable side effects and toxicity, increase drug bioavailability, and target the entrapped drug in the pathological area [26,27,28].

Therefore, we were interested in using niosomes to enhance Oxaliplatin and Paclitaxel anti-colorectal cancer activity and decrease their toxicity. Despite the significant progress in studying the efficiency of niosomes in improving the anticancer activity of the commonly used chemotherapy agents, there are some limitations to measure niosome efficiency in the treatment of colorectal cancer, especially for Oxaliplatin. In spite of the efficacy of niosomes in incorporating hydrophilic drugs, they are still not examined for Oxaliplatin. Previous studies prepared Paclitaxel in a variety of niosome formulations [24,29,30], but they did not consider the efficiency of niosomes in improving anti-colorectal cancer activity.

In this study, we aimed to investigate the effect of using different non-ionic surfactants (Span 60, Tween 80, and TPGS), which were reported for their ability to facilitate the anticancer drugs’ activity [31,32,33,34], in different ratios to formulate nanosized vesicles with accepted Z-potential. These vesicles could be optimized to incorporate both hydrophilic (Oxaliplatin) and hydrophobic (Paclitaxel) colorectal anticancer drugs with high EE%, extended drug release, cytotoxic effect against HT-29 cells, and apoptosis efficiency. To our knowledge, this is considered the first report on comparing the efficacy of niosomes in the incorporation of both hydrophilic and hydrophobic colorectal anticancer drugs.

## 2. Results and Discussion

### 2.1. Drug-Free Niosomes Preparation and Optimization

Niosomes are a promising drug delivery system for cancer therapy as they help in targeting the drug to the cancer cells, increasing the treatment duration with reducing the severe side toxic effects and improving the drug stability [35]. Reducing the particle size and increasing the entrapped drug in the niosomes vesicles improves the drug cytotoxicity in cancer cells [36].

Niosomes were prepared using a thin film hydration method, as it is the most suitable, simple, and reproducible method for the preparation of multilamellar non-ionic niosomal vesicles. It is usually accompanied by sonication to acquire niosomes with a narrow size distribution [37].

Different non-ionic surfactants were used to optimize the drug-free niosomal formulations regarding the particle size and the Z-potential value. CHOL was used in a proper amount to achieve the most stable formulation due to its interaction with non-ionic surfactants, resulting in improvement of the niosomal vesicles’ mechanical strength and permeability to water [38,39], in addition to stability under severe stress conditions [40].

Preparing vesicular carriers with a small particle size was one of the main concerns in this study, as the average size of lipid/nonionic surfactant vesicles is an important parameter with respect to the physical properties and biological fate of niosomes and their entrapped substances [41]. The prepared drug-free formulations had different particle sizes that ranged from 189.2 ± 13.4 nm to 293.3 ± 17.2 nm; see Table 1. The polydispersity index (PDI) of all the prepared niosomes formulations was <0.3, which is considered acceptable for lipid-based vesicles and indicates the formulation homogeneity [42].

**Figure 1:**
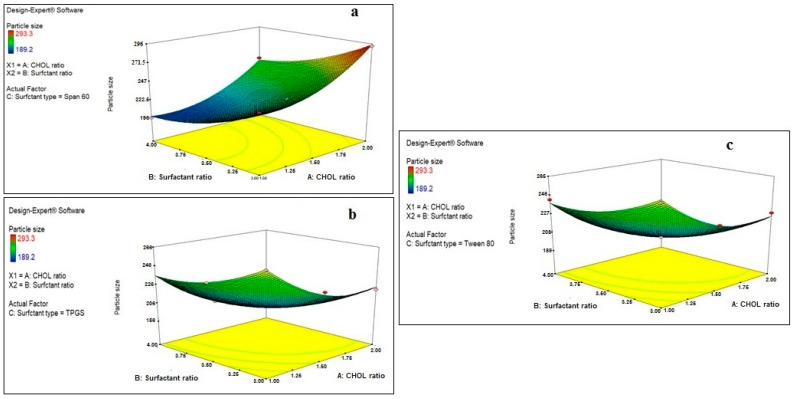
Effect of CHOL and surfactant ratio on the particle size of drug-free niosomes at different surfactant types ((**a**): Span 60, (**b**): TPGS, (**c**): Tween 80).

All the studied factors were found to have a significant effect on the particle size of the prepared drug-free niosomes with significant interaction between the CHOL ratio (X1) and surfactant type (X3), Table 2 and Figure 1; the final equation in terms of coded factors was:

Particle size = +208.82 − 6.05 × A − 17.64 × B + 11.24 × C [1] − 6.42 × C [2] − 1.87 × AB + 32.00 × AC [1] − 6.68 × AC [2] − 3.83 × BC [1] + 0.92 × BC [2] + 16.40 × A^2^ + 9.57 × B^2^

Regarding the effect of the CHOL ratio on the particle size of the prepared drug-free niosomes, a higher CHOL ratio resulted in a significant decrease in the particle size of niosomes formulated with TPGS and Tween 80 surfactants, while it resulted in an increase in the particle size of niosomes formulated with Span 60 surfactant. Cholesterol is an amphipathic rigid molecule with an inverted cone shape, which makes it able to be intercalated between the fluid hydrocarbon chains of the bilayer membrane with its hydrophilic head oriented toward the aqueous surface and aliphatic chain line up parallel to the hydrocarbon chains in the center of the bilayer of vesicles, resulting in increasing the chain order of the liquid-state bilayer and strengthening the nonpolar tail of the non-ionic surfactant [41,43]. For niosomes, the vesicle formation is governed by the hydrophobic interaction between the surfactant and the stabilizing agent, CHOL [44].

Span 60 is known to be more hydrophobic than TPGS and Tween 80, as it has an HLB value of 4.7, while the others have values of 13.2 and 15, respectively. This results in a reduction in the surface free energy associated with the increased lipophilicity [45], which makes Span 60 require less amounts of CHOL to form rigid vesicles. This is in accordance with what was reported previously: that Span 60 could form niosomes either without the addition of CHOL or with small quantities that only maintained the rigidity of niosomes membrane [21]. In addition, with Span 60, higher amounts of CHOL increase the niosomes’ rigidity, which makes them more resistant to the effect of sonication on particle size reduction [46]. Unlike Span 60, TPGS and Tween 80 surfactants require larger amounts of CHOL, which would increase the hydrophobicity and decrease the surface energy, resulting in vesicles with smaller particle sizes. In addition, the hydrogen bonding between the carbonyl group of Tween 80 and the hydroxyl group of CHOL essentially governs the rigidity of the niosomes [47].

A higher surfactant ratio resulted usually in significant lower particle size. This may be related either to the formation of mixed micelles, at higher surfactant amounts, instead of niosomal vesicles, as mixed micelles have lower particle size [48], or to more strengthening of the steric resistance on the vesicle surface due to surfactant adsorption resulting in a lower particle size [49]. TPGS is known to increase the compressibility of the vesicular bilayer as a result of dehydration, when present in high concentrations, and decrease the bilayer defects in the niosomes, resulting in decreasing the particle size [50].

**Figure 2:**
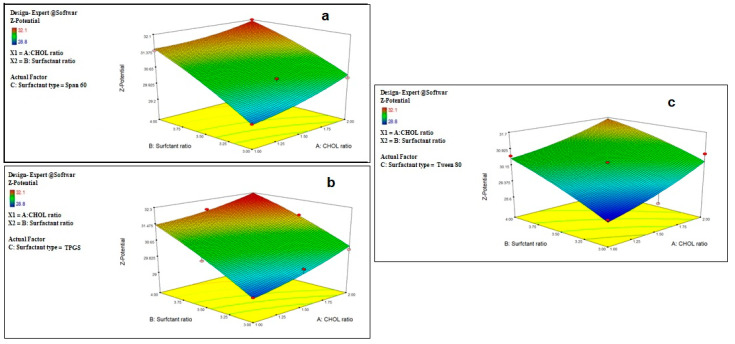
Effect of CHOL and surfactant ratio on the Z-potential of drug-free niosomes at different surfactant types ((**a**): Span 60, (**b**): TPGS, (**c**): Tween 80).

Regarding the effect of different factors on the Z-potential of the prepared drug-free niosomes, both CHOL and surfactant ratio have a positive effect on the Z-potential, while surfactant type has a non-significant effect with non-significant interaction between any two factors, Table 2 and Figure 2, with the final equation in terms of coded factors as:

ZP = +30.61 + 0.55 × A + 0.94 × B + 0.16 × C [1] + 0.17 × C [2] − 0.12 × AB − 0.15 × AC [1] − 7.407 × 10^−3^ × AC [2] + 0.040 × BC [1] + 0.13 × BC [2+ 0.14 × A^2^ − 0.12 × B^2^

Z-potential is an important label for the identification of the prepared nanoparticle physical stability. The system with a Z-potential value around ±30 mV is considered stable [48] due to increasing the repulsion force between the particles, which can overcome the van der Waals attractive forces and hence prevent particles aggregation [51].

Although the prepared niosomes do not include the charge inducer additive, they were found to have accepted negative Z-potential values which ranged from −28.8 ± 2.1 to −32.1 ± 1.9 mV. This might be attributed to the preferential adsorption of hydroxyl ions of the used non-ionic surfactants at the vesicle surface, thus imparting a negative charge to the vesicles surface [41,52,53], and due to the effect of CHOL, as it was reported to impart a negative surface charge on the vesicles’ surface [54]. This can also be related to the surface energy of the vesicles due to the HLB values of the surfactant, as it was reported that an increase in the surface energy of the vesicles leads to an increase in the values of Z-potential toward negative [45].

### 2.2. Optimization of the Prepared Drug-Free Niosomes

**Figure 3:**
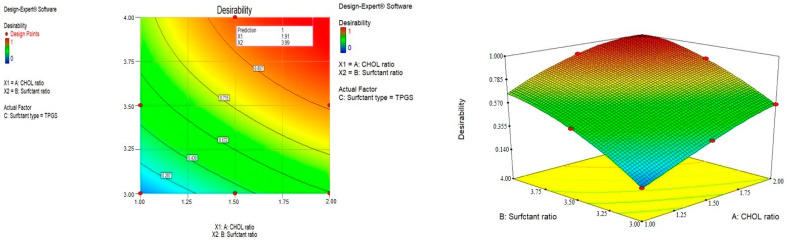
Optimization of the prepared drug-free niosomes.

Responses constraints (particle size was minimized and Z-potential was maximized) were applied to determine the optimum levels of the variables through numerical optimization. The prepared optimized formulations were characterized, and no major residual error was found, indicating the validity of numerical optimization for this study. Different solutions were obtained with the first one having a desirability of 1, as shown in Figure 3, at which the formulation has a particle size of 186.9 nm and a Z-potential of −32.25 mV and consists of TPGS surfactant in a molar ratio of 3.99 with a CHOL molar ratio of 1.91. These results can be represented by F9, which was selected for drug loading. The selected particle size could improve the phagocytosis by macrophages and prolong the plasma drug concentration [55].

### 2.3. Preparation and Evaluation of Drug-Loaded Niosomes

Both drugs, Oxaliplatin and Paclitaxel, could be successfully encapsulated separately into the optimized niosomes formulation with different drug concentrations. To ensure the encapsulation capacity of the prepared niosomes, EE% was determined. As represented in Table 3, increasing the drug ratio from 0.5 to 1 resulted in a significant increase in the EE% for both drugs, with *p* values = 0.0024 and 0.0175 for Oxaliplatin and Paclitaxel, respectively. This was in accordance with reported studies that higher drug concentrations enhance drug entrapment efficiency, as it imparts a driving force for the drug to be encapsulated into the vesicles [56]. There was no difference between the drug concentration in the supernatant before and after the addition of acetonitrile, indicating that there were no niosomes suspended in the supernatant, and all of them were resting in the dialysis bag.

Paclitaxel was significantly entrapped in a higher amount than Oxaliplatin at the same drug ratio, *p* value < 0.05. Theoretically, Paclitaxel, a water-insoluble drug, is placed into hydrophobic tail groups (more hydrophobic drug), while Oxaliplatin is placed in the aqueous core, since Oxaliplatin is more soluble in water. One of the possible reasons for the high entrapped amounts of both drugs might be correlated to the interaction between the drug and the surfactants, which could locate the drug into both hydrophobic tail groups and the aqueous interior part of niosomes.

Further increase in the drug concentration from 1 to 2% did not significantly increase the EE% for both drugs (*p* value = 0.09512 and 0.8297 for Oxaliplatin and Paclitaxel, respectively). This may be attributed to the saturation of the drug within the lipid bilayer of the niosomes, as the excess drug will be scattered between the niosomal pellets and the precipitate [57]. This was also affected by constant concentrations of CHOL and surfactant, which would yield a certain number of vesicles with limited drug loading. This finding indicates the suitability of the selected noisome formulae to encapsulate both hydrophilic and hydrophobic drugs.

The particle size and Z-potential of the prepared drug-loaded niosomes were measured, as shown in Table 3. It was found that increasing the drug concentration from 0.5 to 1 led to a significant increase in the vesicles size (*p* < 0.05), which is in direct correlation with the drug EE%. Drug encapsulation into the niosomal vesicles usually increases their particle size, which may be related to the interaction of the drug with the surfactant head groups, resulting in increasing the charge and mutual repulsion of the surfactant bilayers, thereby increasing the vesicle size [58]. Further increase in the drug concentration did not significantly affect the particle size (*p* > 0.05) of drug-loaded niosomes of either drug. The Z-potential was not significantly changed after loading the niosomes with either Oxaliplatin or Paclitaxel. Depending on these results, drug-loaded niosomes with each drug at a molar ratio of 1 for both drugs were selected for further evaluation. It is worthy here to mention that the PDI values of all the prepared drug-loaded niosomes were less than 0.3, indicating homogenous size distribution.

### 2.4. In Vitro Drug Release

**Figure 4:**
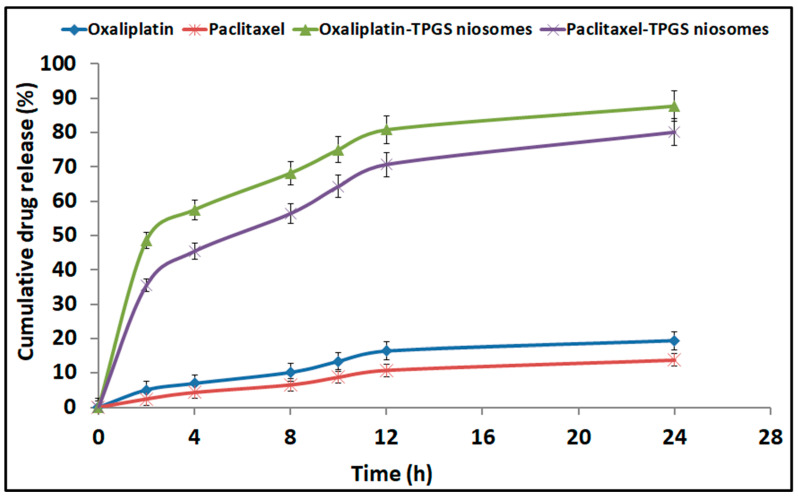
In vitro cumulative drug release profile from Oxaliplatin–TPGS niosomes, Paclitaxel–TPGS niosomes, Oxaliplatin free drug and Paclitaxel free drug.

The release pattern of Oxaliplatin–TPGS niosomes and Paclitaxel–TPGS niosomes in comparison with the free drugs is shown in Figure 4. Both drugs were released from the prepared niosomes at a higher rate than their free drugs. For Oxaliplatin–TPGS niosomes, 87.5 ± 1.99% was released after 24 h compared to 19.4 ± 1.76% from the free drug. For Paclitaxel–TPGS niosomes, 80.81 ± 2.98% was released after 24 h compared to 14.77 ± 0.98% from the free drug. The High EE% and small particle size of the prepared niosomes may be the reason for higher drug release from the prepared niosomes in addition to the hydrophilicity of the TPGS outer shell. The small vesicles size partitions the drug in nanosized particles (<300 nm). In addition, the presence of the surfactant as TPGS, which has a high HLB value and high concentration, facilitated the penetration of release medium to the niosomes surface and into the cores, thus improving the drug release pattern.

The in vitro release pattern of both drugs from the prepared niosomes showed a bi-phasic pattern with an initial burst release followed by sustained release. The high first burst release pattern showed more than 40% at the first 4 h (57.53 ± 0.22% and 45.41 ± 0.43% for Oxaliplatin and Paclitaxel, respectively), which is attributed to the release of the unentrapped and adsorbed drug on the niosomes vesicles’ surface [59]. The second release pattern shows a sustaining release rate for both drugs for 28 h. The significant difference in the second release pattern was due to the bilayered systems such as niosomes, as the drug release occurs by diffusion of the drug from the inner core and passage through the bilayer. In addition, the presence of CHOL, which stabilizes the niosomal bilayer membrane, thus enhances the extended drug release behavior [60]. This sustained behavior of drug release can provide prolonged in vivo drug action while decreasing the dosage frequency.

To determine the effective mechanisms assisting the drugs release from the prepared niosomes formulations, kinetic data were analyzed to express the best fitting mathematical model. Zero-order, first-order, Higuchi diffusion, and Korsmeyer–Peppas models were applied; the correlation coefficients (R^2^) are summarized in Table 4. The best-fit model for both drugs’ release from the prepared niosomes formulations was the Higuchi diffusion model. The latter indicated that the drugs release was a controlled diffusion process based on Fick’s law; i.e., it depends on the time square root. The slow release was previously reported to have a beneficial in reducing the toxic side effects of the entrapped drugs [61,62].

### 2.5. Transmission Electron Microscopy (TEM)

**Figure 5:**
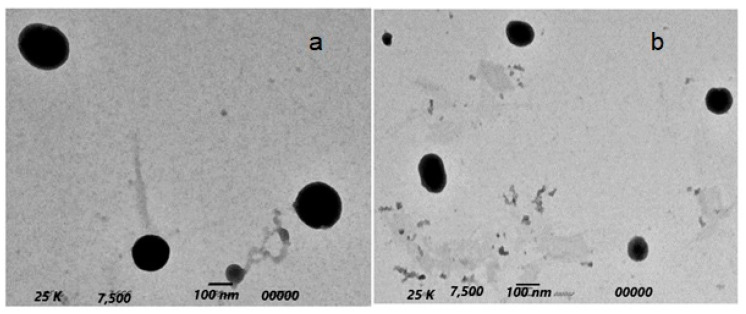
TEM morphology of Oxaliplatin–TGPS niosomes (**a**) and Paclitaxel–TGPS niosomes (**b**).

The morphology of the prepared niosomes is shown in Figure 5. All vesicles had a spherical uni-lamellar morphology with a smooth boundary and homogenous particle size. There was an absence of any aggregation between the nanoparticles, indicating their stability against Oswald ripening by globular collapsing [63].

### 2.6. Evaluation of the Anticancer Activity

#### 2.6.1. Cytotoxicity Study against HT-29 Cells

**Figure 6:**
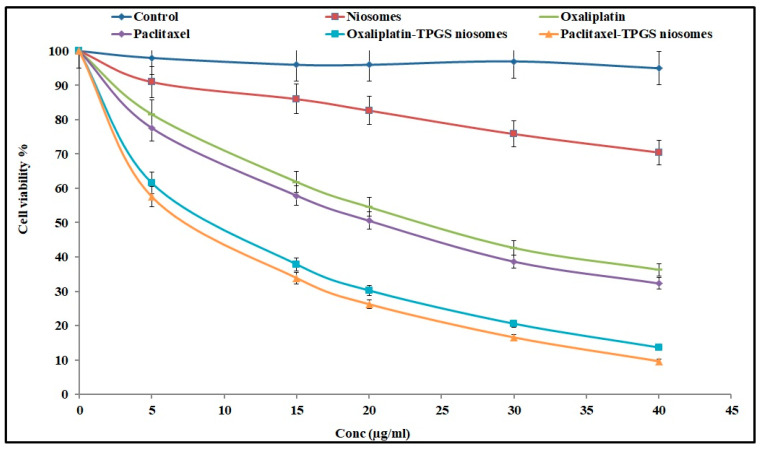
The cytotoxic effect of Oxaliplatin–TPGS–niosomes, Paclitaxel–TPGS–niosomes, drug-free niosomes (F9), Oxaliplatin solution and Paclitaxel solution at various concentrations against HT-29 cells for 24 h (*n* = 3, mean ± SD) (*p* < 0.05).

Oxaliplatin and Paclitaxel were reported for their ability to treat colon cancer. They were tested against HT-29 cells. The cell viability was evaluated by the MTT assay method and compared to the results of plain niosomes and free drugs. The tested formulations (Oxaliplatin–TPGS–niosomes and Paclitaxel–TPGS–niosomes) enhanced their cytotoxicity effect on the colorectal cancer cells. The cytotoxic effect of niosomes in HT-29 cells lines was approximately two-fold compared to that of their free drugs. All tested formulations showed a dose-dependent effect, as shown in Figure 6. The IC_50_ values of Oxaliplatin–TPGS–niosomes, Paclitaxel–TPGS–niosomes, drug-free niosomes (F9), Oxaliplatin solution, and Paclitaxel solution were calculated from the Figure 6 and were found to be 11.86 μg/mL, 7.18 μg/mL, 68.52 μg/mL, 23.56 μg/mL, 19.98 μg/mL, respectively. The significant decrease in the IC_50_ for the prepared niosomes relative to the free drug, about two folds for Oxaliplatin and about three folds for Paclitaxel, is remarkable and indicative of the ability of niosomal formulations to enhance the cellular uptake of both drugs. The significant efficacy of plain niosomes is suggested to be related to TPGS, which is a non-ionic surfactant that has an inhibitory efflux mechanism through ATPase inhibition and subsequent ATP depletion [64,65].

In general, niosomes formulations improved the cancer cell uptake and enhanced the cytotoxicity of both drugs. The high concentration of TPGS enhanced the drug uptake by cancer cells and extended its therapeutic effect. These results are in agreement with what was previously reported: that nanoparticles’ cytotoxic effect is mediated by the internalization and subsequent release of the anticancer drug from nanoparticles intracellularly [64]. There were no significant differences between the cytotoxicity effect represented by IC_50_ value and the cytotoxicity percent of both Oxaliplatin–TPGS–niosomes and Paclitaxel–TPGS–niosomes at the same concentrations (*p* < 0.5). Therefore, niosomes are considered a good targeting carrier system for drug therapy in colorectal cancer for both drugs.

#### 2.6.2. Apoptosis Analysis

**Figure 7:**
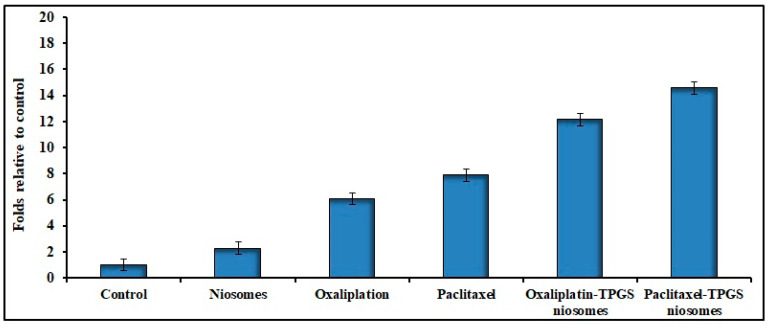
Effects of Oxaliplatin–TPGS–niosomes, Paclitaxel–TPGS–niosomes, drug-free niosomes (F9), Oxaliplatin solution and Paclitaxel solution therapy on apoptosis in HT-29 cancer cell line (IC_50_ values μg/mL) for 24 h treatment in HT-29 cells (*p* < 0.05 compared to control).

The anticancer drugs’ toxicity could be convoluted by apoptosis mechanism [66]. Figure 7 shows the apoptosis result related to the effect of different tested formulations. The apoptotic activity of niosome formulations (Oxaliplatin–TPGS–niosomes and Paclitaxel–TPGS–niosomes) was remarkably higher than that of their free drugs and plain noisome formulation. The noticed free niosomes apoptotic activity was due to the presence of TPGS. It was reported that TPGS can induce cancer cell apoptosis through different mechanisms, either by helping in the destruction and inhibition of the mitochondrial respiratory complex [67] or through induction of DNA damage or oxidation of lipid, protein, and enzyme, leading to cell destruction [68]. This is in agreement with previously reported findings that TPGS has been approved by the FDA as a P-glycoprotein (P-gp) inhibitor, which is an extracellular transporter that influences the pharmacokinetics (PK) of various compounds. Thus, TPGS could enhance the bioavailability and reverse MDR (modified drug release) [66,67,69]. The latter explains the higher niosomes-mediated delivery of the drugs to the cancer cells than the free drugs. It was reported previously that using non-ionic surfactants for niosomes preparation is promising due to their inhibitory effect of p-glycoprotein, which significantly increases the bioavailability of some anticancer drugs [70,71]. The niosomes’ vesicles size also plays an important role in their cell penetration and, consequently, absorption and targeting, as particles with sizes less than 200 nm show higher cellular drug uptake for cancer therapy [72,73]. In addition, the presence of CHOL in the niosomes’ structure could enhance cellular uptake due to the interaction between CHOL and the biological membranes [74]. There was no significant difference between the effect of Oxaliplatin–TPGS–niosomes and Paclitaxel–TPGS–niosomes (*p* < 0.05). These results demonstrate that niosomes represent a promising drug delivery system for anticancer drugs in colorectal cancer therapy. It could also be used to target tumor cells and prolong circulation in the body.

It is worthy here to mention that our results are comparable to the results of previous approaches that have been published about using nanotechnology formulations, other than niosomes, in enhancing the cytotoxic effect and decreasing the side effects of both Oxaliplatin and Paclitaxel. For example, Jabalera et al. formulated Oxaliplatin as biomimetic magnetic nanoparticles (BMNPs), and when they were tested against HT-29 cells, they induced about a two-fold decrease in the IC_50_ value compared to the Oxaliplatin solution [75]. Tummala et al. prepared Oxaliplatin immune-hybrid nanoparticles (OIHNPs) to deliver Oxaliplatin for colorectal cancer treatment, and these nanoparticles resulted in a significant increase in the cellular uptake compared to the free drug when they were tested on HT-29 cells [59]. On the other side, Zhen et al. found that the IC_50_ of Paclitaxel-loaded cationic liposomes synthesized by linoleoyl tails was at least two fold lower than that of cationic liposomes synthesized by oleoyl tails at every tested Paclitaxel content [76].

## 3. Materials and Methods

### 3.1. Materials

Oxaliplatin, Paclitaxel, Cholesterol, Span 60, Tween 80 and d-α-tocopheryl polyethylene glycol 1000 succinate (TPGS) were bought from Sigma-Aldrich Co. (St. Louis, MO, USA). All other chemicals were of analytical grade and were purchased from El-Gomhoria Co., Cairo, Egypt. The chemical structure of the used drugs and non-ionic surfactants is mentioned in Supplementary Material.

The colon cancer cell line HT-29 was cultured in Dulbecco’s modified Eagle’s medium, which contained 4.5 g of glucose per liter and 10% fetal bovine serum (FBS) (Thermofisher Scientific, Waltham, MA, USA). The culture media contained 100 units/mL of penicillin and 100 g/mL of streptomycin. The cells were kept at 37 °C with 5% CO_2_. Prior to treatment with various agents, the cells were cultured in fresh media containing 10% FBS for cell growth and MTT studies.

### 3.2. Experimental Design

To define the optimally selected factors that produce niosomes with minimal particle size and the required Z-potential, response surface D-optimal factorial design was employed to statistically investigate the effect of different formulation variables on the properties of the prepared drug-free niosomes using Design-Expert^®^ software (version 7; Stat-Ease, Inc., Minneapolis, MN, USA). Three independent factors were screened at three different levels as follows: cholesterol (CHOL) molar ratio (X1) at 3, 3.5, and 4, surfactant molar ratio (X2) at 1, 1.5, and 2, and surfactant type (X3) at Span 60, TPGS and Tween 80. Two independent variables were evaluated, which were particle size (Y1) and Zeta potential (Z-potential) (Y2). The design parameters and constraints are shown in Table 5, and their detailed composition is reported in Table 5.

### 3.3. Preparation of Drug-Free Niosomes

Drug-free niosomes were prepared by a thin film hydration method [20,77]. Nonionic surfactants (Span 60, TPGS and Tween 80) and CHOL were accurately weighed separately and dissolved in 10 mL chloroform then transferred to a round-bottom flask. The residual solvent was allowed to evaporate under reduced pressure using a rotary evaporator (Rotavap, Type R-110, Buchi, Switzerland) at 150 rpm and 65 °C for 2 h until the formation of a thin lipid film on the inner flask wall. After thin film formation, the dried film was then hydrated using 10 mL phosphate buffer saline pH 7.4 pre-heated to 65 °C with rotation for 1 h until dispersion was obtained. The dispersion was left to equilibrate at 25 °C overnight and then subjected to sonication using a probe sonicator (Sonifier^®^ 250 Branson, MO, USA) in an ice-bath for three intermitted intervals, each one for 5 min. Dispersions were kept in a tightly closed container at 4 °C for evaluation.

### 3.4. Particle Size and Z-Potential Analysis

The particle size, estimated by dynamic light scattering (DLS), and Z-potential of the prepared drug-free niosomes were measured by a Zeta-sizer (Zeta-sizer Ver. 7.01, Malvern Instruments, Worcestershire, UK) after appropriate dilution of the samples with de-ionized water (1:10) to avoid multi-scattering phenomena using standard operation methods. All measurements were conducted in triplicate at 25 ± 1 °C. Results were recorded as the mean ± SD.

### 3.5. Preparation of Drug-Loaded Niosomes

Based on statistical optimization of the prepared drug-free niosomes, one formulation having the minimal particle size and maximum Z-potential was selected to be loaded separately with the two drugs: Oxaliplatin and Paclitaxel. Drug-loaded niosomes were prepared in the same method as the drug-free niosomes (Section 3.3), while the drug was added in different amounts: 0.5, 1, and 2 molar ratios. Paclitaxel was dissolved in the organic phase (10 mL chloroform), while Oxaliplatin was dissolved in the pre-heated phosphate buffer saline pH 7.4 (65 °C).

### 3.6. Evaluation of the Prepared Drug-Loaded Niosomes

#### 3.6.1. Drug Entrapment Efficiency (EE%)

The drug entrapment efficiency in the prepared drug-loaded niosomes was determined using the dialysis technique against phosphate-buffered saline (PBS, pH 7.4) for separation of the non-entrapped drug from the niosomes dispersion [78,79]. From each formulation, 3 mL of niosomal suspension was dropped into a dialysis bag (M.Wt. cut off: 12000. Medicell, London, UK). The bag was immersed into a beaker containing 100 mL phosphate-buffered saline (PBS, pH 7.4) with constant stirring at 4 °C. After every 30 min, samples were withdrawn, and the concentration of the free drugs was measured spectrophotometrically (Schimadzu spectrophotometer, Model UV-1601, Marsiling Industrial Estate, Singapore) at 260 nm and 227 nm for Oxaliplatin and Paclitaxel, respectively. Dialysis was complete when no more drugs were detectable in the recipient solution. The percentage of drug entrapment in the drug-loaded niosomes was calculated according to the following equation [80].

Drug Entrapment % = [(Total Drug − Drug in the supernatant)/Total Drug] × 100

All the measurements were calculated three times, and results were represented as mean ± SD.

For confirmative studies, 1 mL of acetonitrile was added to 5 mL of supernatant with stirring to lyse any present niosomes into the supernatant. The solution was filtered, properly diluted with PBS (pH 7.4), and the drugs concentration was measured spectrophotometrically.

#### 3.6.2. Measurement of the Particle Size and Z-Potential of Drug-Loaded Niosomes

The mean vesicles size and Z-potential value of the prepared drug-loaded niosomes formulations were calculated as previously described in Section 3.4. Results were recorded as the mean ± SD.

#### 3.6.3. In Vitro Drug Release Study

To study the release pattern of both drugs from the prepared drug-loaded niosomes, an in vitro release study was performed using the dialysis bag method applying the sink conditions [22]. Two milliliters of either Oxaliplatin–TPGS niosomes or Paclitaxel–TPGS niosomes were placed in a dialysis bag of 50 mm flat width and 10 k Da, MWCO. The both-ended closed bag was placed in a conical flask containing 150 mL PBS pH 7.4 containing 1% sodium lauryl sulfate as a medium. The whole assembly was shaken using a thermostatically controlled shaker (PSU-20i Orbital Multi-Platform Shaker, Grant Instruments (Cambridge) Ltd., Thomas Scientific, Swedesboro, NJ, USA) at 37 °C and 50 rpm. Samples were withdrawn at 2 h intervals for 24 h and immediately replaced with pre-heated fresh medium to maintain the sink conditions. The cumulative amount released was determined spectrophotometrically at 260 nm and 227 nm for Oxaliplatin and Paclitaxel, respectively, and the cumulative amount released was calculated. The same method was repeated with the drug-free niosomes to be used as a blank. For comparative study, the release pattern of both pure drugs separately was studied in the same method. All measurements were calculated three times and results were represented as mean ± SD. Different models as zero-order, first-order, Higuchi diffusion, and Korsmeyer–Peppas were applied to evaluate the drug release pattern and determine the kinetics model that expresses the drug release mechanism from the prepared formulations [81].

#### 3.6.4. Transmission Electron Microscopy (TEM)

Morphological examination of the optimized Paclitaxel–niosomes and Oxaliplatin–niosomes was conducted using Transmission Electron Microscope (TEM) (JEOL JEM1230, Tokyo, Japan). A drop of each formulation was placed on a carbon-coated copper grid to leave a thin film, which was negatively stained with 1% phosphotungstic acid (PTA). The grid was left to dry, and samples were scanned under the transmission electron microscope operating at an accelerating voltage of 80 kV.

### 3.7. Evaluation of the Anticancer Activity for the Selected Paclitaxel-Niosomes and Oxaliplatin-Niosomes

#### 3.7.1. Cytotoxicity Study against HT-29 Cells

The cytotoxicity study on HT-29 (human colon adenocarcinoma) cells using an MTT (tetrazolium salt 3-[4,5-demethylthiazol-2-yl]-2-5-diphenlytetrazolium bromide) colorimetric method was completed for the following formulations: Oxaliplatin–TPGS niosomes, Paclitaxel–TPGS niosomes, drug-free niosomes (F9); Oxaliplatin and Paclitaxel solutions were used as positive controls. HT-29 cells were seeded in 96-well plates at a density of 5 × 10^3^ cells and then incubated for 24 h at 37 °C. The tested cells were treated with series concentrations of the tested formulations (all containing an equivalent concentration) separately for 24 h at 37 °C. Cell viability was evaluated with MTT on a Synergy 2 Multi-Detection Microplate Reader by BioTek Instruments, Inc at 570 nm. Six independent experiments were conducted, and the inhibitory concentration (50%) (IC_50_) was determined. Results were expressed as mean ± SD compared to the negative control of untreated cells (100% proliferation) [82].

#### 3.7.2. Cell Apoptosis and Cell Cycle Assay of HT-29 Cells

The TUNEL method was used to analyze the ability of the selected niosomal formulations to induce apoptosis in HT-29 cells. The following formulations: Oxaliplatin–TPGS niosomes, Paclitaxel–TPGS niosomes, and drug-free niosomes (F9) were tested; Oxaliplatin and Paclitaxel solutions were used as positive controls. Sigma plot software was used to obtain the best-fit straight line, and the cellular apoptosis was expressed in folds relative to control cells (the untreated cells). The cells were seeded in 6-well plates and were treated with IC_50_ values of all the tested treatments and incubated for 24 h [83]. Six independent experiments were conducted, and results were expressed as mean ± SD.

### 3.8. Statistical Analysis

All data were expressed as the mean of triplicate ± standard deviation (SD). The formulation design and evaluation were performed using the Design-Expert 13^®^ Software, Version 13.2.03, 2021, Stat-Ease, USA. One-way ANOVA was applied to assess the formulation factors’ effect on the selected niosomes formulations characters considering *p* ≤ 0.05 statistically significant.

## 4. Conclusions

Different formulation variables could be optimized to obtain niosomal vesicles having a low particle size and an accepted Z-potential. Optimized niosomes prepared by the thin-film hydration method using TPGS surfactant in a molar ratio of 4 along with cholesterol in a molar ratio of 2 were loaded with either Oxaliplatin or Paclitaxel in different molar ratios, and those with a molar ratio of 1 resulted in the highest EE% values, 90.57 ± 2.05 and 93.51 ± 2.97, respectively. Delivering both drugs as vesicular niosomes helped in modifying their release rate compared to their free drugs, as they showed extended drug release, which could lead to a decrease in their toxicity. The encapsulation of Oxaliplatin and Paclitaxel into the niosomes particles markedly enhanced their cytotoxicity effect along with apoptosis efficiency up to two to three fold compared to their free drugs. Therefore, niosomes preparation using non-ionic surfactant with certain anti-colorectal cancer activity as TPGS could be considered a unique nanomicellar system for high encapsulating and delivering hydrophilic drug such as Oxaliplatin and hydrophobic drug such as Paclitaxel with improving their therapy outcomes against colorectal cancer, taking into consideration cost effect.

## Figures and Tables

**Table 1:** Experimental runs, independent and dependent variables of the factorial experimental design of drug-free niosomes.

Runs	Factors (Independent Variables)			Responses (Dependent Variables)		
	CHOL Ratio (*w*/*w*)	Surfactant Ratio (*w*/*w*)	Surfactant Type *	Particle Size (nm)	Z-Potential (mV)	PDI
F1	1.00	3.00	Span 60	242.5 ± 22.4	(−) 29.3 ± 1.8	0.158 ± 0.01
F2	1.00	4.00	Span 60	198.2 ± 18.6	(−) 31.4 ± 1.6	0.214 ± 0.04
F3	1.50	3.25	Span 60	232.1 ± 15.7	(−) 30.4 ± 2.1	0.256 ± 0.11
F4	2.00	3.00	Span 60	293.3 ± 17.2	(−) 30.2 ± 1.7	0.247 ± 0.21
F5	2.00	4.00	Span 60	251.2 ± 20.3	(−) 32.1 ± 1.9	0.165 ± 0.06
F6	1.00	3.00	TPGS	265.3 ± 18.4	(−) 29.1 ± 1.7	0.146 ± 0.04
F7	1.00	3.50	TPGS	241.2 ± 16.7	(−) 30.2 ± 2.2	0.132 ± 0.03
F8	1.50	3.00	TPGS	231.5 ± 18.2	(−) 29.8 ± 2.4	0.189 ± 0.14
F9	2.00	4.00	TPGS	194.4 ± 15.5	(−) 31.8 ± 1.9	0.175 ± 0.20
F10	2.00	3.00	TPGS	221.2 ± 21.3	(−) 30.2 ± 1.6	0.211 ± 0.07
F11	2.00	3.50	TPGS	198.1 ± 17.8	(−) 31.5 ± 1.8	0.241 ± 0.31
F12	1.00	4.00	Tween 80	241.7 ± 19.8	(−) 30.6 ± 2.4	0.257 ± 0.45
F13	1.00	3.00	Tween 80	261.4 ± 22.6	(−) 28.8 ± 2.1	0.237 ± 0.25
F14	1.50	3.00	Tween 80	228.3 ± 19.5	(−) 28.9 ± 1.5	0.198 ± 0.41
F15	1.50	3.50	Tween 80	203.1 ± 17.9	(−) 30.3 ± 1.8	0.269 ± 0.09
F16	2.00	4.00	Tween 80	189.2 ± 13.4	(−) 31.5 ± 2.2	0.222 ± 0.17
F17	2.00	3.00	Tween 80	228.4 ± 16.4	(−) 30.7 ± 2.1	0.243 ± 0.29

* Surfactant type; hydrophilic–lipophilic balance (HLB) value: Span 60 (HLB;4.7), TPGS (HLB; 13.2), Tween 80 (HLB; 15).

**Table 2:** The design expert results of all response variables.

Source	Particle Size (nm)		Z-Potential (mV)	
	F	*p*-Value	F	*p*-Value
Model	68.78	<0.0001	12.67	0.0058
A: CHOL ratio	23.40	0.0047	33.52	0.0022
B: Surfactant ratio	236.62	<0.0001	92.89	0.0002
C: Surfactant type	31.81	0.0014	3.40	0.1168
AB	1.86	0.2313	1.17	0.3288
AC	178.48	<0.0001	0.78	0.5055
BC	3.05	0.1359	0.85	0.4817
A^2	49.58	0.0009	0.56	0.4872
B^2	12.94	0.0156	0.32	0.5939
Adequate precision	29.912		12.934	
R^2^	0.9934		0.9654	
Adjusted R^2^	0.9790		0.8892	
Predicted R^2^	0.8413		0.5883	
SD	4.11		0.34	
%CV	1.78		1.12	

**Table 3:** Effect of drug concentration on EE%, particle size, and Z-potential of the prepared drug-loaded niosomes.

Drug Loaded (Molar Ratio)	Oxaliplatin–TPGS Niosomes				Paclitaxel–TPGS Niosomes			
	EE%	Particle Size (nm)	Z-Potential (mV)	PDI	EE%	Particle Size (nm)	Z-Potential (mV)	PDI
0.5	77.19 ±2.68	236.4 ± 22.3	−31.7 ± 0.96	0.236 ± 0.07	83.82 ± 3.13	227.4 ± 16.3	−30.91 ± 0.45	0.283 ± 0.04
1	90.57 ±2.05	278.5 ± 19.7	−32.7 ± 1.01	0.264 ± 0.05	93.51 ± 2.97	251.6 ± 18. 1	−31.69 ± 0.98	0.273 ± 0.08
2	91.03 ±2.80	285.8 ± 23.5	−33.25 ± 1.41	0.295 ± 0.07	93.31 ± 3.31	258.6 ± 13.3	−32.99 ± 1.08	0.287 ± 0.09

**Table 4:** Different mathematical models of in vitro release data (means ± SD, *n* = 3).

Formulation	Correlation Coefficient (R^2^)			
	Zero Order	1st Order	Higuchi Diffusion	Korsmeyer–Peppas
Oxaliplatin–TPGS–niosomes	0.6114 ± 0.034	0.8715 ± 0.027	0.8871 ± 0.021	0.8844 ± 0.011
Paclitaxel–TPGS–niosomes	0.7164 ± 0.021	0.9006 ± 0.014	0.9475 ± 0.011	0.942 ± 0.015

**Table 5:** Factorial design of drug-free niosomes composition.

Factors	Levels		
	Low (−1)–High (1)		
A (X1): Cholesterol (molar ratio)	3	3.5	4
B (X2): Surfactant (molar ratio)	1	1.5	2
C (X3): Surfactant type	Span 60	TPGS	Tween 80
**Responses**			
(Y1): Particle size (PS)	Minimize		
(Y2): Zeta potential (Z-potential)	Maximize		

## Data Availability

Data is contained within the article and supplementary material.

## Supplementary Materials

The following supporting information can be downloaded at: https://www.mdpi.com/article/10.3390/ph15070816/s1.
