# Supplementary material for: Targeting Colorectal Cancer Cells with Niosomes Systems Loaded with Two Anticancer Drugs Models; Comparative In Vitro and Anticancer Studies
Source: Pharmaceuticals (Basel). 2022 Jun 30;15(7):816. doi: 10.3390/ph15070816 (PMC9323826; doi:10.3390/ph15070816)
Supplement: Supplementary file 1 [file pharmaceuticals-15-00816-s001.zip › pharmaceuticals-1781793-supplementary.pdf]

The used drugs

Oxaliplatin

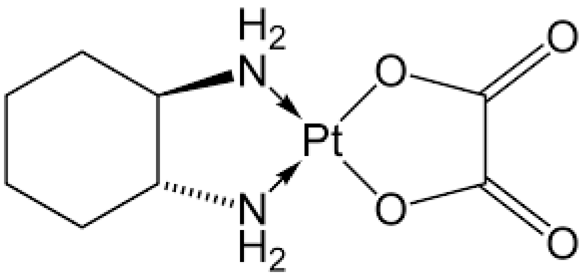

(1R, 2R)-2-azanidylcyclohexyl]azanide;oxalic acid;platinum(2+)

Paclitaxel

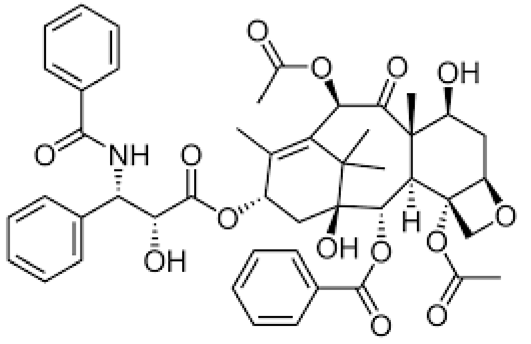

(1S,2S,3R,4S,7R,9S,10S,12R,15S)-4,12-diacetyloxy-15-[(2R,3S)-3-benzamido-2-hydroxy-3-phenylpropanoyl]oxy-1,9-dihydroxy-10,14,17,17-tetramethyl-11-oxo-6-oxatetracyclo[11.3.1.03,10.04,7]heptadec-13-en-2-yl] benzoate

The used non ionic surfactant

Span 60

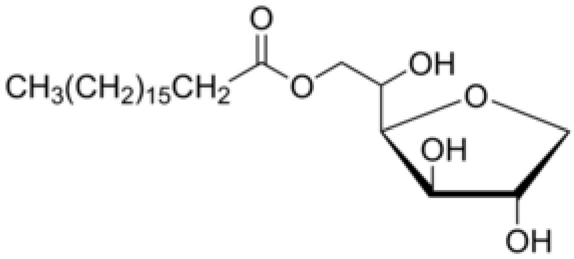

(2R)-2-[(2R,3R,4S)-3,4-dihydroxyoxolan-2-yl]-2-hydroxyethyl] octadecanoate

Tween 80

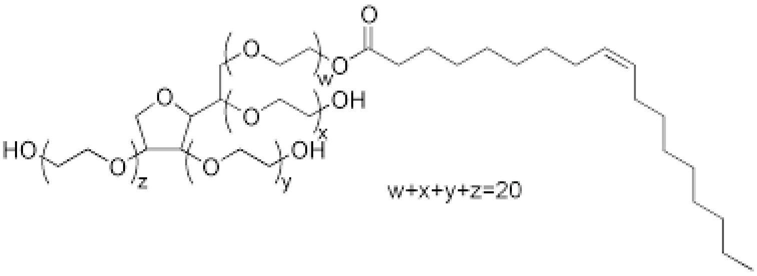

2-{2-[3,4-bis(2-hydroxyethoxy)oxolan-2-yl]-2-(2-hydroxyethoxy)ethoxy}ethyl (9E)-octadec-9-enoate

d-α-tocopheryl polyethylene glycol 1000 succinate (TPGS)

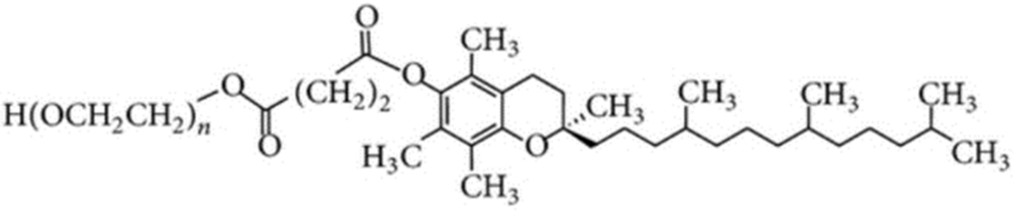

MONO-(2,5,7,8-TETRAMETHYL-2-(4,8,12-TRIMETHYLTRIDECYL)-6-CHROMANYL) SUCCINATE POLYETHYLENE GLYCOL MONOESTER
